# Culturally Adapting a Digital Intervention to Reduce Suicidal Ideation for Syrian Asylum Seekers and Refugees in the United Kingdom: Protocol for a Qualitative Study

**DOI:** 10.2196/47627

**Published:** 2023-06-22

**Authors:** Oliver Beuthin, Kamaldeep Bhui, Ly-Mee Yu, Sadiya Shahid, Louay Almidani, Mariah Malak Bilalaga, Roshan Hussein, Alnarjes Harba, Yasmine Nasser

**Affiliations:** 1 Department of Psychiatry University of Oxford Oxford United Kingdom; 2 Department of Theoretical and Applied Linguistics University of Cambridge Cambridge United Kingdom; 3 Wilmer Eye Institute Johns Hopkins University School of Medicine Baltimore, MD United States; 4 Medstar Health Baltimore, MD United States; 5 n/a London United Kingdom; 6 Centre for Global Health St George's, University of London London United Kingdom

**Keywords:** cultural adaptation, digital mental health, suicidal ideation, refugee mental health, Syrian refugee, experience-based co-design, mental health, suicide, suicidal, refugee, immigrant, ethnic minority, asylum, user experience, cultural, Syria, Syrian, refugees

## Abstract

**Background:**

The conflict in Syria has produced the largest forced displacement crisis since the Second World War. As a result, Syrians have experienced various stressors across the migratory process, putting them at an increased risk of developing mental health issues, including, crucially, suicidal ideation (SI). Despite their high rates of SI across Europe, there remain various barriers to accessing treatment. One way to increase access is the use of culturally adapted digital interventions, which have already shown potential for other minority populations. To culturally adapt the intervention, further research is needed to better understand Syrian asylum seekers’ and refugees’ cultural conceptualizations, coping strategies, and help-seeking behavior for SI. To do so, this study will use a unique cultural adaptation framework to intervene at points of lived experience with the migratory process where Syrian culture and signs of psychopathology converge. Likewise, co-design events will be used to adapt points of experience with the intervention where Syrian culture and the intervention conflict. As the first cultural adaption of a digital SI intervention for Syrian asylum seekers and refugees, this study will hopefully encourage further development of culturally sensitive interventions for the largest refugee population in the United Kingdom and the world.

**Objective:**

The objective of the study is to increase access to mental health treatment for Syrian asylum seekers and refugees in the United Kingdom by culturally adapting a digital intervention to reduce SI.

**Methods:**

The study will use experience-based co-design, an action research method, to culturally adapt a digital intervention to reduce SI for Syrian asylum seekers and refugees in the United Kingdom. This will involve conducting 20-30 interviews to understand their lived experiences with the migratory process, cultural conceptualizations of mental health and SI, coping strategies, mental health help-seeking behavior, and perceptions of digital mental health interventions. In addition, 3 co-design events with 6 participants in each will be held to collaboratively adapt the intervention. Touchpoints and themes extracted from each phase will be prioritized by a community panel before adapting the intervention.

**Results:**

The study began in November 2022 and will continue until the last co-design event in August 2023. The results of the study will then be published by December 2023.

**Conclusions:**

Access to treatment for some of the most severe mental health issues is still limited for Syrian asylum seekers and refugees in the United Kingdom. Cultural adaptations of digital interventions developed for general populations have the potential to increase access to treatment for this population. Specifically, adapting the intervention for Syrian asylum seekers’ and refugees’ experiences with SI in relation to their lived experience with the migratory process may enable greater recruitment and adherence for users of various cultural and ethnic subgroups and levels of SI.

**International Registered Report Identifier (IRRID):**

DERR1-10.2196/47627

## Introduction

### Background

Since 2011, the civil war in Syria has resulted in the loss of over half a million lives and the largest forced displacement crisis since the Second World War [1]. By recent estimates, this includes 6.7 million displaced within Syria and 6.8 million refugees living abroad [2]. Of those displaced, over 1 million Syrians have reached Europe. As asylum seekers and refugees, they are likely to experience high rates of mental health issues, including major depressive disorder, posttraumatic stress disorder, and suicidality [3]. It is therefore important to improve the understanding and care of mental health for Syrian asylum seekers and refugees arriving in the United Kingdom.

### Suicidal Ideation

One of the most concerning mental health issues for asylum seekers and refugees across Europe is suicidal ideation (SI), with research showing high rates in Sweden, 33.9% [4] and 48% [5]; Switzerland, 42.1% [6] and >30% [7]; and the United Kingdom, 40.9% [8]. Asylum seekers’ and refugees’ elevated rates of SI may be explained by their experiences of the migratory process [9]. While in their countries, they are often exposed to bombings, torture, and bereavement [1,3,10]. The journey to their first country of asylum often involves encountering human traffickers [11], unsafe modes of transport including inflatable boats and closed containers [12], and harsh living conditions in refugee camps [13]. Upon arriving in the first country of asylum, asylum seekers may face punitive immigration control policies [12] or a prolonged asylum application process [14]. After receiving refugee status, they may continue to experience stressors associated with a loss of social networks and environmental mastery [3,15,16], with acculturation, with discrimination, and with mental health services based on dominant models of illness in Western developed countries [17]. Thus, in order to develop acceptable and effective treatments, more research is needed to understand the specific determinants of SI for asylum seekers and refugees, and in particular Syrians, the largest refugee population globally and in the United Kingdom.

Despite asylum seekers’ and refugees’ elevated risks for SI, they still underuse mental health services [18-21]. Some of the barriers to accessing treatment include a lack of knowledge about mental health services, a lack of culturally competent treatment, fears about confidentiality, limited proficiency in English, and logistical issues such as transport difficulties and immigration status [21]. Again, further research is needed to understand the specific barriers and facilitators of mental health help-seeking among Syrian asylum seekers and refugees in the United Kingdom.

### Cultural Adaptation

As a complex problem, reducing SI among asylum seekers and refugees in the United Kingdom will inevitably require complex structural interventions. However, reducing SI can start with the short-term individual adoption of simple clinical interventions [22]. Specifically, treatment for SI among Syrian asylum seekers and refugees in the United Kingdom may be improved with culturally adapted digital mental health interventions [23,24], which already seem promising for ethnic minorities [25], refugees in general [26,27], and Syrian refugees specifically [28].

Cultural adaptation involves “the systematic modification of an evidence-based treatment...or intervention...protocol to consider language, culture, and context in such a way that it is compatible with the client’s cultural patterns, meanings, and values” [29]. Based on the universality of emotional expression, Bernal and colleagues’ [29] framework of cultural adaptation asserts that while mental health beliefs and help-seeking behavior differ across cultures, the distinct signs and symptoms of psychopathology are universal. They therefore hold that cultural adaptations should attempt to maintain the integrity of the original intervention as much as possible. Conversely, Horrell [30] asserts that rather than adapting existing interventions, entirely new interventions should be developed for different cultural groups. Although her framework is more situated, it still does not acknowledge the dynamic nature of culture within cultural groups. These contrasting frameworks can perhaps be integrated by identifying the signs of psychopathology through shared experiences that give rise to core affect, including hedonic valence and physical arousal, and conceptual knowledge [31]. Thus, an embodied and experience-based approach to emotions will allow one to use changes in experience [32] to predict a person’s core affect and core beliefs [33]. Importantly, where cognitive behavioral therapy (CBT) commonly involves challenging distorted core beliefs through cognitive reconstruction, in some circumstances, mental health issues may arise as a result of experiential challenges to a person’s core beliefs, such as the inherent value of one’s life and safety. In which case, challenges to the core belief could result in identity crisis [33] and suicidal behavior [34]. Thus, the cultural adaptation process can be simplified by focusing on points of lived experience with the migratory process at which signs of psychopathology such as SI and Syrian culture coincide [35]. In addition, due to Syrian education policies, Syrian ethnic and linguistic groups are only able to read and write in Modern Standard Arabic [35]. Therefore, based on a lived experience paradigm, the intervention will be adapted for Modern Standard Arabic but will identify and adapt marked language use. A possible research approach to simplify the cultural adaptation process is experience-based co-design (EBCD) [36]. Specifically, this will entail identifying crucial moments, known as touchpoints, that make a difference in Syrian asylum seeker and refugee experiences with the migratory process such as their experiences of SI in response to torture or the asylum process. After culturally adapting the intervention using the themes and touchpoints identified during interviews, Syrian asylum seekers and refugees will participate in co-design events to collectively identify and adapt touchpoints of the intervention at which Syrian culture and the intervention’s core mechanisms of change conflict.

### Digital Intervention

This study will build on Eylem and colleagues’ [24] cultural adaptation of the Leven onder Controle (literally “Living under Control”) intervention, a guided 6-week CBT-based digital intervention to reduce SI developed by van Spijker and colleagues [37]. The intervention includes 6 modules: thinking about suicide, dealing with thoughts and feelings, thinking about the future, thinking about the self, thinking about others, and repetition and relapse. Randomized controlled trials of the intervention have found it to be effective in reducing SI compared to treatment as usual—an information website—for general Dutch (*d*=0.2) [38] and Belgian populations (*d*=0.34) [39], but not for Australian populations [40].

Based on Bernal and colleagues’ [29] cultural adaptation framework, Eylem and colleagues [24] adapted the intervention for Turkish immigrants in the United Kingdom and the Netherlands by including cultural metaphors, treatment concepts, and goals, as well as the context and methods of the intervention. Although largely successful in culturally adapting the intervention, Eylem and colleagues [24] were unable to recruit enough participants for a full randomized controlled trial. They therefore suggested a need for further optimization of the recruitment process and intervention including culturally appropriate strategies for recruitment, identifying the determinants of SI, and a diversity of case studies both in terms of culture and SI severity.

### Aims

This research aims to improve the understanding and treatment of SI among Syrian asylum seekers and refugees arriving in the United Kingdom. As the research has shown varying rates of SI among asylum seekers and refugees [4-8], lived experience data will help to identify the determinants of SI across the migratory process and specifically for Syrian asylum seekers and refugees in the United Kingdom. Moreover, EBCD will help simplify the cultural adaptation process and increase access to treatment for SI for a greater number of Syrian cultural, ethnic, and linguistic subgroups.

### Objectives

The first phase of the study will involve one-to-one semistructured interviews to (1) identify touchpoints across the 3 stages of migration (pre-, peri-, and postmigration); (2) explore how Syrian refugees conceptualize, cope with, and seek help for mental health and SI; (3) identify cultural, linguistic, and structural barriers and enablers for seeking help for mental health and SI; and (4) understand Syrian refugee perceptions of digital mental health interventions. The second phase will include community panel discussions and co-design events to (5) prioritize touchpoints and themes extracted from interview data with a community panel to adapt the intervention; (6) conduct co-design events to identify touchpoints to culturally adapt the intervention; and (7) prioritize touchpoints and themes from the co-design events with the community panel to adapt the intervention.

## Methods

### Study Design

In this study, an accelerated version of EBCD will guide the cultural adaptation process of a digital intervention to reduce SI for Syrian asylum seekers and refugees in the United Kingdom. EBCD is an action research method for collaboratively improving health care services and interventions [36]. Although traditionally used to improve existing services, EBCD has more recently been used to incorporate users’ lived experiences of mental health problems into digital interventions [41]. For example, Lewis and colleagues [41] used EBCD to develop a mental health mobile app for health care workers affected by COVID-19. They conducted photo interviews with up to 30 health care workers to understand their mental health needs and lived experiences. The photos were used to draw out touchpoints related to health care workers’ experiences during the pandemic and the effects on their mental health. Touchpoints were then further explored during co-design workshops to develop content for the app. Similarly, this study will involve semistructured interviews to gather Syrian asylum seeker and refugee lived experiences with the migratory process. Given the traumatic nature of some asylum seeker and refugee experiences, the 3 phases of migration, pre-, peri-, and postmigration, will be used to structure interviews and draw touchpoints instead of using photos. The interviews will also explore their cultural conceptualizations, coping strategies, and help-seeking behavior for mental health and SI, as well as their perceptions of digital mental health interventions. The study will be accelerated by focusing data collection on specific stakeholders, Syrian asylum seekers and refugees in the United Kingdom, rather than mental health professionals whose role was more important during the initial development of the intervention. Instead, touchpoints and themes identified in the interviews will be prioritized by a community panel during group meetings before adapting the intervention in preparation for co-design events. The community panel will consist of researchers, mental health professionals, and cultural brokers associated with the Syrian community in the United Kingdom. Similar to Lewis and colleagues’ [41] study, the cultural adaptation process will include co-design events where participants will be presented with the partially adapted intervention and collectively identify and adapt touchpoints related to the intervention. The use of co-design events to empower participants to directly culturally adapt the intervention is another methodological innovation of the study, with previous attempts having only involved participants indirectly [24]. Again, the community panel will meet after touchpoints and themes are extracted from the co-design events to balance any need for cultural adaptation with maintaining the integrity of the intervention’s core mechanisms of change.

### Setting

As Syrian asylum seekers and refugees are dispersed across the United Kingdom, interviews and co-design events will be held on the web to lower barriers to participation.

### Participants

In total, 40-50 male or female Syrian asylum seekers and refugees in the United Kingdom, aged 18 years and older will participate in one-to-one semistructured interviews and 3 focus groups (co-design events). Recruitment objectives include 20-30 participants for interviews and 18 participants for 3 focus groups with 6 participants in each group. The participants will include different genders (male and female), age groups (18-39 years and 40 years and older), ethnicities (Arab, Kurdish, and other), religious groups (Muslim, Christian, and other), and refugee status (asylum seeker or refugee). Previous research has shown that most of the information is revealed in the first 5-6 interviews [42]; however, due to the stigma associated with suicide, participants may underreport their experiences with SI. Therefore, 20-30 participants will be recruited to increase the likelihood of identifying participants with experiences of SI. Likewise, a minimum level of SI will not be set for inclusion but rather individuals with serious SI, defined as a score of 4 and above on the SI subscale of the Columbia-Suicide Severity Rating Scale, will be excluded from participation [43,44]. This decision was made to avoid risks of harm to the participants and has thus far not resulted in the exclusion of any participants. To reduce the time spent on recruitment, interview participants will be asked to participate in a co-design event focus group.

### Sampling

The study will use purposive sampling to recruit participants from various demographic backgrounds including age, gender, religion, ethnicity, and refugee status. Participants will be recruited via correspondence with nongovernmental organizations and charities and adverts on social media. At the time of writing, 16 participants have already been recruited through Syrian cultural brokers with no participants from NGOs or charities. The main barrier to recruitment through NGOs and charities has most often been low capacity. As suggested by Eylem and colleagues [24], cultural brokers can increase recruitment capacity where certain organizations produce an institutional barrier for subgroups of migrants [45]. In fact, this may be true for all Syrians who, due to government-sanctioned repression, have understandably developed a mistrust for authority structures in general [46].

### Data Collection

#### Lived Experience Interviews

The first phase of the study will involve one-to-one semistructured interviews with Syrian asylum seekers and refugees in the United Kingdom. The interviews will follow a topic guide including open-ended questions about participants’ lived experience with the migratory process and the effects it has had on their mental health; their cultural and personal conceptualizations of mental health and SI; the ways in which they cope with mental health and SI; the barriers and facilitators for seeking help for mental health and SI; and their perceptions of digital mental health interventions (programs). Given the stigma attached to mental health [21], semistructured interviews that focus on Syrian refugees’ mental health in relation to their experiences with the migratory process are more likely to encourage participation and also reduce researcher bias. Touchpoints will be extracted from the interviews to better understand the mechanisms by which SI arises for this population and provide culturally relevant and appropriate case examples in the intervention. Previous cultural adaptation work on this intervention conducted by Eylem and colleagues [24] found case examples over emphasized SI and traditional values. Therefore, focusing on Syrian asylum seekers’ and refugees’ experiences with the migratory process should provide a shared paradigm for a culturally diverse population with varying levels of SI. Since asylum seekers and refugees are dispersed across the United Kingdom, interviews will be conducted remotely using Microsoft Teams or over the phone. Prior to conducting the interviews, participants will provide oral consent, which will include consenting to being audio-recorded and indirectly quoted in any publications. Participants’ consent will be recorded by the researcher on an oral consent form, and only digital copies will be kept by the researcher and participant. Digital case report forms will also be used to collect data about the participants age, gender, ethnicity, religion, refugee status, and general practitioner registration. Subsequently, the interviews will be conducted for 30-45 minutes, which will be voice recorded and then transcribed and translated into English.

#### Co-Design Events

The co-design events will involve collecting data through 3 focus groups with 6 participants in each. This will involve bringing participants of diverse backgrounds together to identify key touchpoints of the intervention for cultural adaptation. The adaptation will build on Eylem and colleagues’s [24] Turkish cultural adaptation of van Spijker and colleagues’ [37] 6-week guided CBT-based web-based intervention to reduce SI. Prior to the focus group, participants will be given access to 2 modules that have already been translated and partially adapted. Modules will be paired based on the order in which they are provided in the intervention. The 2 modules will be presented again at the beginning of each focus group. Subsequently, participants will be asked to identify the main touchpoints and ways to adapt the modules. The focus groups will be conducted on the web using Microsoft Teams to facilitate participation from various locations across the United Kingdom. Prior to conducting the focus groups, participants will provide oral consent in the same manner as the interviews. Digital case report forms will also be used to collect demographic data including age, gender, ethnicity, religion, refugee status, and general practitioner registration. The focus group will then be conducted for no longer than an hour and a half, which will also be voice recorded, transcribed verbatim, and translated into English for analysis.

### Data Analysis

Semistructured interviews will be transcribed verbatim, and thematic analysis will be used to identify themes, as well as touchpoints of participants’ lived experience with the migratory process to culturally adapt the intervention. Thematic analysis was chosen for its epistemological flexibility [47]. Analysis will combine both realist and social constructionist paradigms. Braun and Clarke [47] describe thematic analysis as identifying semantic themes for specific areas of interest in the data or latent themes spread across the data. Moreover, they assert that semantic themes are revealed through description, whereas latent themes only arise after interpretation. However, the distinction between semantic and latent meaning disregards the primary roles of intentions and context in meaning interpretation and construction [48]. In other words, what is obvious in one context may be latent in another and vice versa, hence, the need for cultural adaptation. For example, mental health issues may be latent in discussions about mental health but obvious in discussions about lived experiences with the migratory process. Additionally, mental health issues may be latent during the premigration phase and more obvious in the postmigration phase. Thus, to combine both epistemologies, the analysis will be inductive but will be bound by the objectives of the study. The code system and themes will be developed in accordance with the 6 phases outlined by Braun and Clarke [47] and will be checked by another member of the research team. The stages include data familiarization, generating initial codes, searching for themes, reviewing themes, defining and naming themes, and producing the report. Internal reliability will be ensured by an iterative review of themes as well as checking for consistency between members of the research team [49]. Where irreconcilable differences in interpretations arise, preference will be given to the interpretation in line with participants’ shared lived experience and the objectives of the study. Such differences are likely to arise due to an analyst’s background knowledge either as a researcher or from lived experience. Therefore, adherence to the objectives of the study and the participants’ lived experiences in analysis is as essential to the cultural adaptation process as adherence to the intervention’s mechanisms of change and participants’ lived experience is to maintaining implementation fidelity. Internal validity will be ensured by iteratively checking for correspondence between the thematic map and the data set [49]. Given the study involves semistructured qualitative data, this will involve comparing the thematic map to the original codes before structuring them into themes. The use of a thematic map to guide the analysis is also important as NVivo, a qualitative analysis software produced by Lumivero, mostly supports the representation of linear and not complex relationships. The co-design events will also help to determine the credibility of the analysis through participant validation of lived experience case examples, coping strategies, and help-seeking behavior [49]. The co-design event data will be analyzed using the same methods, and themes will be prioritized by a community panel before the final adaptation of the intervention.

### Ethics Approval

Ethics approval has been provided for the study by the Central University Research Ethics Committee at the University of Oxford (R81752/RE001).

### Informed Consent

Eligible participants will receive a participant information sheet for either an interview or focus group. This will describe, in both Arabic and English, the goals and limitations of the study, the way interviews and focus groups will be performed, the way data will be collected and handled, and any risks involved in participating. Participants will be informed that they are free to withdraw from participation at any time, for any reason, without consequence, and are not obliged to provide the reason for their withdrawal. The contents of the participant information sheet will be explained verbally by the DPhil researcher, OB, or one of the members of the research team before conducting the interview or focus group, and participants will have the opportunity to ask questions. Oral informed consent will then be obtained from participants before screening for inclusion and exclusion criteria and participating in an interview or focus group. Informed consent will be recorded on an oral informed consent form on behalf of the participant, and digital copies will be given to the participant and kept on university servers for at least 3 years from the time of publication.

### Safety Protocol

Although the study will not necessarily involve the recruitment of participants directly experiencing SI, if OB suspects that any participants are at risk of harming themselves or in need of help, he will encourage them to contact their local general practitioner and provide them with information for alternative sources of help. Likewise, participants will be informed in the participant information sheet that confidentiality could be broken where there is a risk of harm to themselves or others. In which case, OB will report any risk of harm to the principal investigator, KB, who will in turn review the circumstances and act according to his judgment. If there is a risk of immediate harm, the emergency services will be contacted via 999. If further steps are necessary, the matter will be referred to the Multi-Agency Safeguarding Hub within 1 working day of first receiving the information from the participant. The local area designated officer will then advise on the next appropriate steps to take.

### Data Management

Access to personal data will be restricted to only authorized individuals. Personal names will be replaced with unique participant numbers on consent forms and audio recordings and transcriptions of interviews. Focus group audio recordings and transcriptions will be given a unique focus group number and individual electronic case report forms will be labeled with the participant’s initials. All audio recordings will be deleted once they have been transcribed. The remaining data will be kept on university servers for 3 years from the time of first publication in accordance with Oxford University policy and then deleted by the principal investigator.

### Dissemination

The findings of this study will be disseminated through publication and web-based Syrian asylum seeker and refugee communities. Likewise, the feasibility and acceptability of the culturally adapted intervention will be assessed in a later study as part of OB’s dissertation research.

## Results

The first phase of the study, lived experience interviews, was initiated in November 2022. Since commencing, 16 participants have been recruited and interviewed. The second phase of the study, co-design events, will commence in June 2023. The study, including the write-up period, is expected to end in December 2023.

## Discussion

### Expected Findings

This paper describes the study protocol for the cultural adaptation of a digital SI intervention for Syrian asylum seekers and refugees in the United Kingdom. The main aim of the study is to improve the understanding and treatment of SI among Syrian asylum seekers and refugees arriving in the United Kingdom. Accordingly, the study will consist of two phases: (1) semistructured interviews and (2) co-design events. First, interviews will be conducted to explore the lived experiences of Syrian asylum seekers and refugees in the United Kingdom with the migratory process; their mental health and SI conceptualizations, coping strategies, and help-seeking behavior, including cultural, linguistic, and structural barriers; and their perceptions of digital mental health interventions. This integrative approach to cultural adaptation identifies lived experience as the source of both psychopathology and their subsequent cultural interpretations and help-seeking. Specifically, this includes touchpoints, crucial moments that make a difference to Syrian asylum seeker’ and refugee’ experiences with the migratory process. Importantly, touchpoints can highlight the ways implicit boundaries of lived experience such as refugee status impact mental health. Second, co-design events will be held to enable Syrian asylum seekers and refugees to collaboratively and culturally adapt the intervention. While digital evidence-based interventions have generally been effective in reducing SI, most studies have revealed low adherence rates, with participants completing only half of the intervention [50-52]. Thus, rather than evaluating intervention outcomes, the study will help achieve greater access to treatment for this population by optimizing the recruitment process and the intervention.

### Limitations

While the cultural adaptation of a digital mental health intervention for Syrian asylum seekers and refugees is aimed at reducing barriers to treatment for SI, it nonetheless will not affect their ongoing lived experiences with the migratory process. It is therefore hoped that digital mental health interventions will help Syrian asylum seekers and refugees in the United Kingdom cope with the asylum process, which will often involve reopening the wounds caused by their experiences of conflict in Syria. It is also important to acknowledge the difficult ethical dilemma of balancing the need to explore their lived experiences and the effects it might have on their mental health. This study is therefore indebted to the Syrian asylum seekers and refugees who have already shared their experiences with the expectation that this research will be used for the benefit of their community. While digital SI interventions may have limited capacity for systems change, their most substantial contribution is to help preserve the lives and mental health of those who must live in them. The study design is also limited to culturally adapting the digital intervention; however, its feasibility and acceptability to Syrian asylum seekers and refugees in the United Kingdom experiencing SI will be assessed in a future study.

### Conclusions

The conflict in Syria has resulted in the largest forced displacement crisis since the Second World War [1]. As a result, Syrian asylum seekers and refugees have been exposed to several stressors throughout the migratory process, including dangerous journeys and a prolonged asylum process [9]. Their experiences have put them at an increased risk of developing mental health issues including crucially SI [3]. Despite their elevated rates of SI, Syrian asylum seekers and refugees often fail to seek treatment due to language barriers, the lack of culturally competent services, mental health stigma, and the fear of deportation [18-21]. Thus, access to treatment for SI among Syrian asylum seekers and refugees in the United Kingdom may be improved with culturally adapted digital mental health interventions [23]. In this study, this will involve using EBCD, an action research method for collaboratively improving health care services and interventions, to culturally adapt a digital SI intervention developed for the general population [36]. Syrian asylum seekers and refugees in the United Kingdom will be interviewed to explore their cultural conceptualizations, coping strategies, and help-seeking behavior for SI in relation to their lived experiences with the migratory process. They will then collaboratively and culturally adapt the intervention during 3 co-design events to reduce any negative consequences of the cultural adaptation and improve engagement. Cultural adaptation frameworks have traditionally consisted of various components [29], and therefore, EBCD was chosen for its focus on experience touchpoints. Touchpoints of lived experience will not only provide signs of psychopathology behind cultural interpretations and responses but points at which both interact. Co-design events will also enable participants to identify shared touchpoints in the intervention in a similar way to their lived experience with the migratory process. Studies that include opportunities for co-design not only facilitate engagement but could also increase mental health literacy among their target population. Finally, it is hoped that the cultural adaptation of a digital SI intervention for Syrian asylum seekers and refugees in the United Kingdom provides an example of procedural fairness in facilitating equal access to mental health care.

## Abbreviations

CBT: cognitive behavioral therapy
EBCD: experience-based co-design
SI: suicidal ideation

## Data Availability

The data sets generated during and/or analyzed during this study are not publicly available due to the vulnerable nature of the participants but are available from the corresponding author on reasonable request.
